# Profile of caregivers of Parkinson’s disease patients and burden measured by Zarit Scale Analysis of potential burden-generating factors and their correlation with disease severity

**DOI:** 10.1590/1980-57642018dn12-030011

**Published:** 2018

**Authors:** Paulo Eduardo Mestrinelli Carrilho, Marcelo Alvarez Rodrigues, Brenda Camila Reck de Oliveira, Emanuelle Bianchi da Silva, Taline Alisson Artemis Lazzarin Silva, Leticia da Silva Schran, Marcia Mendes

**Affiliations:** 1 Assistant Professor. Neurology & Neurosurgery Department - Medicine School of State University of Western Paraná (Unioeste), Cascavel, PR, Brazil.; 2 Resident Preceptor. Neurology & Neurosurgery Department - Medicine School of State University of Western Paraná (Unioeste), Cascavel, PR, Brazil.; 3 Medicine Student. Medicine School of State University of Western Paraná (Unioeste), Cascavel, PR, Brazil.; 4 Nurse. Nursery School of State University of Western Paraná (Unioeste), Cascavel, PR, Brazil.; 5 Senior Nurse and Assistant Professor - Nursery School of State University of Western Paraná (Unioeste), Cascavel. PR, Brazil.

**Keywords:** Parkinson’s disease, disease severity index, caregivers, assistance, doença de Parkinson, índice de gravidade da doença, assistência, cuidadores

## Abstract

Parkinson’s disease (PD) promotes burden among patients and caregivers.

**Objective:**

To analyze whether disease severity (UPDRS and Karnofsky index), total disease duration, patient cognitive status (MMSE), presence of other diseases, patient age, socioeconomic conditions (ABEP2015), living together with patient, total time caregiving, weekly hours of care and presence of assistance from other caregivers are correlated with, and influence statistically, the degree of caregiver burden measured by the Zarit Burden Interview (ZBI).

**Methods:**

After ethics Committee approval, patients and respective caregivers were recruited. Following evaluation with the proper scales, all data were submitted to Pearson’s correlation method and multivariate linear regression analysis (ANOVA).

**Results:**

A total of 21 patients and respective caregivers were evaluated. 72% (N=15) of caregivers reported burden. One third of caregivers reported a moderate or severe level of burden. A cause-effect relationship could not be established by the statistical method adopted, but disease severity measured by the UPDRS was the sole variable showing statistically significant moderate positive Pearson’s correlation with ZBI (r=0.48, for p<0.05). On ANOVA, however, no independent variable had a statistically significant impact on ZBI scores.

**Conclusion:**

Despite our conflicting results, optimization of the available treatment, with better control of PD severity, can be considered an important element to effectively achieve the goal of reducing burden among caregivers.

## Resumo

A doença de Parkinson (PD) promove sobrecarga entre pacientes e cuidadores.

**Objetivo:**

Analisar se a severidade da doença (UPDRS e Karnofsky index), o tempo total de doença, as condições cognitivas do paciente (MEEM), a presença de outras doenças, a idade do paciente, as condições socioeconómicas (ABEP2015), o fato de se ambos vivem juntos, o tempo total que o cuidador exerce esta função, as horas semanais de cuidados e a presença de assistência de outros cuidadores poderiam ser correlacionados e influenciar estatisticamente o grau de sobrecarga dos cuidadores, medido pela entrevista de Zarit (ZBI).

**Métodos:**

Após a aprovação do comitê de ética, o recrutamento de pacientes e respectivos cuidadores foram feitos e após a avaliação com as escalas adequadas, todos os dados foram submetidos ao método de correlação de Pearson e análise de regressão linear multivariada (ANOVA).

**Resultados:**

Avaliou-se um total de 21 pacientes e respectivos cuidadores. 72% (N=15) de cuidadores reportaram ter sobrecarga. Um terço deles relatou nível moderado ou severo de sobrecarga. Não foi possível estabelecer relação causa-efeito pelo método estatístico adotado, porém a severidade da doença, medida pelo UPDRS, foi a única variável que alcançou correlação de Pearson positiva moderada com significância estatística com a ZBI (*r*=0,48, com *p*<0,05), mas na ANOVA, nenhuma variável independente teve um impacto estatisticamente significante nas pontuações de ZBI.

**Conclusão:**

Apesar dos nossos resultados conflitantes, a otimização do tratamento disponível, com melhor controle da severidade PD, poderia ser considerada como um elemento importante e que poderia efetivamente atingir o objetivo de reduzir a carga entre os cuidadores.

Parkinson’s disease (PD) is characterized by motor and non-motor disturbances (psychosis, cognitive impairment, depression, among others) that often promote patient burden but also require care from relatives of the PD patient.^1^ The majority of care for patients with PD is provided by informal caregivers.^2^^,^ 3 Their caregiving not only offers physical and emotional support for patients but also plays a major economic role and prevents early nursing home placement.^2^^,^ 3 To support caregivers in this role, it is necessary to understand the extension of caregiver-burden and factors associated with increased caregiver-burden and distress.^2^^,^ 3 It seems natural to imagine that there is an emotional overload with a burden that apparently can have an extensive negative impact on the quality of life of these caregivers, with consequences even for patients. Many scales and structured instruments for investigating caregiver burden were developed and studied, including in Brazil. Glozman’s Scale of Quality of Life of Caregivers,^4^ the Neuropsychiatric Inventory Caregiver Distress scale (more suitable instrument for evaluating caregiver burden in behavioral disorders),^5^ the Caregiver Burden Scale,^6^ the Family Burden Interview Schedule^7^ and Caregiver Burden Inventory^8^ are listed, among many others. In this sense, the Zarit Burden Interview (ZBI)^9^ is one of the simpler and easy-to-use scales of this group. The ZBI is considered one of the most widely used scales for burden assessment in caregivers of elderly patients with dementia and related disorders.^9^ Despite being developed for clinical and research purposes, with a focus on elderly patients with dementia and their relatives, ZBI items are comprehensive and deal with dimensions common to several mental and physical illnesses. Therefore, the ZBI has been used to assess the burden of caregivers of elderly patients with dementia and of elderly people and adults with other mental and physical diseases, such as stroke survivors, individuals who have chronic illnesses and caregiver burden of subarachnoid hemorrhagic patients. The ZBI has been translated into several languages and is used in many countries besides the US, where it was originally developed. It has also been translated and validated in Portugal^10^ and Brazil^11^ with different objectives.

Despite the importance of the problem and the existence of many available instruments to quantify and study burden in caregivers of PD patients, well-designed articles about the real utility of objective scales for quantification of burden among PD caregivers are relatively scarce, compared to other lines of research in PD. The objectives of such studies are generally related to establishing a correlation between the severity of caregiver burden and other variables that may possibly and significantly interfere with the magnitude of the burden score obtained by these structured interviews. Studies on this topic are of fundamental importance to elucidate the diverse aspects of this problem. Anxiety and depressive disturbances, emotional distress, complaints about economic burden are all examples of these aspects related to caregiver burden. They are frequently neglected, since there is usually an absolute focus of attention on the patient and their disease. The relevance of caregiver problems and their quality of life are commonly overlooked by professional teams involved with these patients. Such studies can also disclose new perspectives on the development of new instruments and management strategies aimed at creating innovative solutions to the problems arising from caregiver burden.

The aim of the present study is to describe our experience with ZBI among PD patient caregivers and to correlate, using statistical methods, ZBI scores with some presumed or probable burden-generating factors in caring for PD patients, naming, disease severity, disease duration, patient cognitive status, presence of other patient co-morbidities, patient age, whether caregiver lives with the patient and time dedicated to patient care, socioeconomic conditions and, finally, the presence or absence of a “helping hand” from other caregivers, sharing some care tasks in the direct management of the patient.

## METHODS

The study was initiated after approval by the Ethics Committee on Medical Research and after signing of the consent form for participation in the studies both by patient and related caregiver. Data collection for evaluations and scales applied in both groups of patients and their respective caregivers occurred between July 2015 and June 2016, attended in the outpatient care clinic for parkinsonian patients of the State University of the West of Paraná (UNIOESTE), Brazil. The eligibility criteria were: patients with Parkinson’s disease diagnosed by a neurologist trained in “movement disorders”, based on the criteria of the “UK Parkinson Disease Society Brain Bank”.^12^ The caregivers were recruited according to the patient’s decision and after their designation about who was the main caregiver. The sole criterion for exclusion was the presence of serious or sufficiently severe morbidities to constitute a major bias in statistical analysis in both patients and caregivers. The severity assessment of patients with PD was performed using 2 standard scales (Unified Parkinson’s Disease Rating Scale^13^ - UPDRS - and the Karnofsky index^14^ - KI). Data were also collected on duration of the disease, existence of pre-existing diseases and current cognitive status (Mini-Mental State Exam^15^ - MMSE). The caregivers were subjected to evaluation of burden using the ZBI. ZBI score is related to burden level. The 22 ZBI items reflect respondent’s areas of concern such as: health, social and personal life, financial situation, emotional well-being and interpersonal relationships. Items measure the objective and subjective burden reported by the caregiver, but these scores are not obtained separately. The subjective way in which items are written favors the caregiver’s emotional answer. The scale’s last item is a general one, in which the respondent is asked to assess to what extent they feel burdened by their caregiver role. Higher scores are taken to indicate increased burden intensity (score: ≤21=no burden; 21-40 = mild burden; 41-60 = moderate burden; ≥61 = severe burden).^11^

Data on socioeconomic conditions were collected, using the classification according to the 2015 survey of the Brazilian Association of Marketing and Publicity (ABEP 2015).^16^ The data on the presence or absence of support by others in caregiving and the total time as caregiver and weekly hours dedicated to the patient were obtained. Information on whether or not the caregiver resides with the patient and on the relationship or kinship between them was also collected. The ZBI was applied to all 21 caregivers. It was a systematized interview that could be answered by caregiver alone or, if necessary, conducted by one of the investigators.

The data were analyzed in a descriptive epidemiological statistical method using Pearson’s correlation coefficient (*r*) test. The relationship between the scores obtained on the ZBI and the other independent variables was verified through simple and multivariate linear regression analysis of variance (ANOVA), with a confidence interval of 95%. The values obtained for ZBI scores were studied by this method along with several other variables that could be subjected to descriptive statistical analysis to determine which of these could be more related to an increase in the burden measured by the ZBI and ascertain the content of this relationship.

The Pearson’s correlation coefficient test (*r*) correlated the following variables in relation to the values obtained for ZBI scores: UPDRS scores (patient), KI scores (patient), data on disease duration (patient), current cognitive status of patient, number of comorbidities (patient), patient age, whether patient and caregiver share the same dwelling, weekly hours dedicated (caregiver), time giving care in months/years (caregiver), socioeconomic classification according to the ABEP2015 (caregiver), and help (or not) from others (caregiver). The magnitude of the values of the Pearson’s correlation coefficient (*r*) were classified using Dancey and Reidy^17^ criteria as follows: (independent of sign): *r*=0.10 up to 0.30 (weak correlation), *r*=0.40 up to 0.60 (moderate correlation) and *r*=0.70 up to 1 (strong correlation). Statistical significance was considered for p<0.05.

## RESULTS

A total of 21 patients and their respective caregivers were studied, giving 42 evaluations overall. Among the caregivers, 80% were female and 47% had more than 8 years of education. The average age was 53 years (SD= ±12.44). The total time as carer ranged from 6 months to 30 years (median=9.5 years). In the patient group, mean age was 67.95 years (SD=±13.70). In relation to the degree of kinship with the patient, 47% (n=10) were partners, 38% (n=8) son or daughter, 5% (n=1) grandchild; 5% (n=1) parent and 5% (n=1) daughter-in-law. Regarding caregivers, most (72% - n=15)respondents lived in the same house as the patient and 62% (n=13) received assistance in the task of caring. The task was performed in general by son or daughter (24% - n=5) or brother/sister (24% - n=5). The socioeconomic classification of caregivers, according to the ABEP 2015 criteria, revealed a profile of predominantly classes B2 (33% - n=7) and C1 (29% - n=6). The time in years that the caregiver had performed the role was 12 years on average (minimum = 6 months / max = 24 years) and, at the time of the survey, time dedicated to care averaged 43 hours per week (minimum = 2 / max = 168). The chart presented displays data collected from caregivers and respective patients (Table 1).

**Table 1:** Data on caregivers and respective patients.

Caregiver number and gender	Patient initials and gender	Caregiver age	Patient age	Relationship	Patient educational level	PD duration (years)	Patient ocupation	Same home	Weekly hours of care	Help/who provides	Other diseases (number)	ABEP2015	MMSE	KI	UPDRS	ZBI
1 - F	A.B. - M	36	60	Daughter	Illiterate	9	Retired	Yes	35	Yes- mother	No	C1	24	60	18	7
2 – F	E.C. – F	30	68	Daughter-in-law	Primary	6	Retired	Yes	7	No	Yes (4)	B2	25	80	39	24
3 – F	T.C.L. – F	48	71	Daughter	Illiterate	15	Unemployed	Yes	84	Yes – sister	No	B1	29	60	42	30
4 – F	E.M.F. – F	58	89	Daughter	Illiterate	19	Retired	Yes	42	Yes-brother	Yes (1)	C2	10	40	40	28
5 – F	A.P.Z.- M	67	74	Partner	Secondary	6	Retired	Yes	84	No	No	C1	26	70	37	5
6 – F	V.F. – M	66	71	Partner	Primary	15	Retired	Yes	98	No	Yes (1)	C2	25	60	40	16
7 – M	J.S.- M	36	64	Son	Illiterate	19	Unemployed	No	10	No	Yes (1)	B2	18	60	49	34
8 – F	D.M.- F	57	83	Daughter	Illiterate	8	Retired	No	14	No	Yes (1)	C2	23	60	41	45
9 – M	C.M.D. – M	51	80	Son	Illiterate	35	Retired	No	8	Yes-brother	No	D OR E	15	50	35	56
10 – F	G.L. – M	42	65	Daughter	Illiterate	10	Farmer	No	21	Yes – sister	No	B1	27	80	14	43
11 – M	I.M.B.- F	30	75	Grandson	Illiterate	2	Retired	No	4	Yes-brother	Yes (2)	B2	28	100	6	6
12 – F	R.Z. – M	40	71	Daughter	Primary	3	Unemployed	Yes	168	Yes-brother	Yes (1)	B2	17	40	76	44
13 – F	G.C. – M	71	79	Partner	Illiterate	20	Retired	Yes	14	No	Yes (1)	C1	16	80	36	12
14 – F	R.H. – M	43	47	Partner	Higher	9	Retired	Yes	42	Yes – son	Yes (1)	B2	30	60	53	33
15 – F	I.R. – M	56	61	Partner	Illiterate	16	Retired	Yes	14	Yes-daughter	Yes (1)	B2	23	80	28	24
16 – M	P.K. – M	71	42	Father	Primary	7	Retired	No	2	Yes-partner	No	C1	29	70	45	14
17 – F	S.G.A.- M	72	74	Partner	Illiterate	9	Retired	Yes	140	Yes-children	Yes (1)	B2	29	80	30	28
18 – F	J.C.B- M	66	79	Partner	Primary	17	Retired	Yes	14	No	Yes (2)	D OR E	20	60	11	27
19 – F	O.N.T.P.-M	56	43	Partner	Secondary	15	Retired	Yes	14	Yes-children	No	C1	30	80	45	35
20 – F	I.R.S.- M	54	67	Partner	Illiterate	1	Operative worker	Yes	14	Yes-children	Yes (3)	C1	21	60	67	62
21 - F	A.C. - M	63	64	Partner	Primary	4	Retired	Yes	84	No	Yes (1)	B2	20	60	26	23

ABEP2015: Socioeconomic classification according to Brazilian Marketing Association 2015 version (A – highest level to E – lowest level); MMSE: Mini-Mental State Exam scores; KI: Karnofsky index scores; UPDRS= Unified Parkinson’s Disease scores; ZBI= Zarit Burden Interview scores.

**Figure 1:**
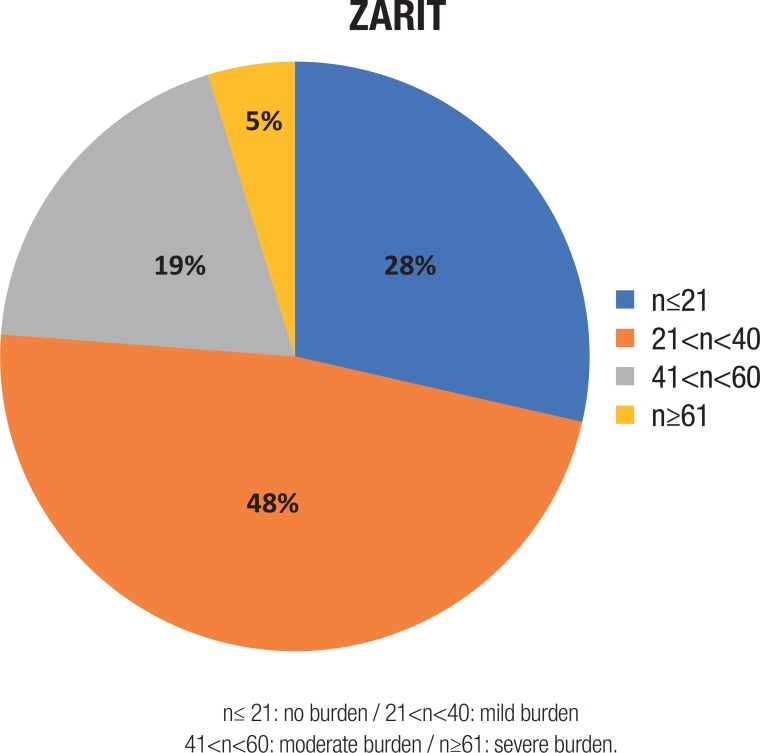
ZBI score distribution among caregivers n≤ 21: no burden / 21<n<40: mild burden 41<n<60: moderate burden / n≥61: severe burden.

The scores obtained on the ZBI ranged from 7 to 62, where 28% (n=6) of patients scored ≤21, 48% (n=10) 21-40, 19% (n=4) 41-60 and 5% (n=1) >61. The percentage pie diagram in the article shows the distribution of ZBI scores among caregivers (Figure 1).

Results of Pearson’s correlation tests (*r*) and *p*-value (ANOVA F of significance in simple linear regression analysis) between the dependent variable (ZBI) and independent variable are listed below:

**Table:** 

**Figure 2:**
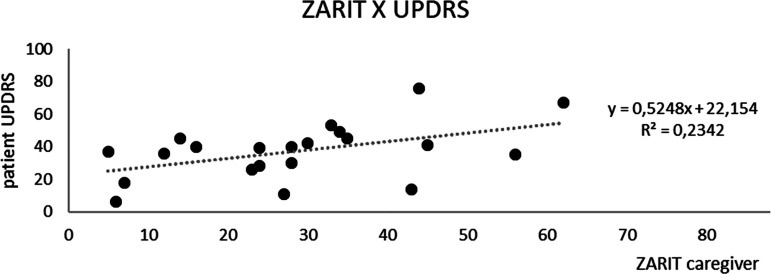
Scatter plot: ZBI (caregiver Zarit) and UPDRS patient scores with linear regression analysis and trendline plus R^2^ calculation.

• ZBI and UPDRS *r* =0.48 (moderate positive correlation) with *p*-value=0.0262*
• ZBI and KI *r*= –0.42 (moderate positive correlation) with *p-*value=0.0570
• ZBI and disease duration *r*=0.18 (negligible correlation) with *p-*value=0.4298
• ZBI and MMSE *r*= –0.25 (weak negative correlation) with *p*=0.2651
• ZBI and other morbidities *r*=0.12 (weak positive correlation) with p=0.6091
• ZBI and patient age *r*=0.08 (negligible correlation) with *p*=0.7180
• ZBI and residing in same house *r*= –0.19 (negligible correlation) with *p=*0.4076
• ZBI and hours dedicated to care *r*= –0.04 (negligible correlation) with *p*=0.8694
• ZBI and total time as carer *r*=0.02 (negligible correlation) with *p=*0.7475
• ZBI and ABEP classification *r=* –0.1 (negligible correlation) with *p=*0.6730
• ZBI and help from others *r*=0.26 (weak positive correlation) with *p*=0.2948


* *p*<0.05 – see scatter plot with trendline and R^2^ (Figure 2).

When analyzed by multivariate linear regression of variance (ANOVA), no independent variable reached statistical significance (*p<*0.05). The results are given in the ANOVA table (Table 2).

## DISCUSSION

Regarding PD, quality of life is a major concern and has been extensively studied using different methods.^18^^-^ 20 Likewise in Brazil, many articles^20^^-^ 22 address this problem. Schetasky^22^ et al., studying quality of life among caregivers of PD patients, used the WHOQOL-BREF administrated face-to-face by an experienced psychiatrist. The WHOQOL-BREF questionnaire consists of 26 items, whose application time is approximately 5-10 minutes. The ZBI is considered a simple interview method dispensing with the need for a formal investigator. However, in spite of this consideration, an investigational agent was responsible for applying the Zarit interview in the present survey. The study disclosed 72% burden in PD patient caregivers (48% mild, 19% moderate and 5% severe burden). Most caregivers were first-degree relatives (partner and/or children), similar to other studies.^19^^,^ 23 Most caregivers lived together with the patient, probably reflecting a peculiarity of Brazil, where few institutions for geriatric care exist for patients who intend to reside within an institution. Most caregivers also received consistent help from other relatives in caregiving. The stratification according to socioeconomic conditions revealed that the distribution of caregivers was representative of the local population. Female gender was predominant among caregivers (about 80%), perhaps reflecting the role of family caregivers, traditionally provided by women in rural regions similar to the study setting, where social relationships remain archaic compared to urban groups in larger cities of Brazil. Another observation is that only two patients were still working and 76% were retired, maybe reflecting the advanced age of this group, perhaps associated with the disability imposed by PD. This observation might be related, to some extent, with the need for some sort of care from others.

**Table 2:** Influence of all variables on ZBI scores. Multivariate linear regression analysis (“ANOVA”).

	Coefficients	Standard error	Stat t	P–value
Intersection	–1.1622513	63.24320726	–0.01838	0.985739
UPDRS	0.363516819	0.296613001	1.225559	0.251467
MMSE	0.677025084	1.135074897	0.596459	0.565568
Disease duration	0.696595041	0.932580117	0.746955	0.474152
Patient age	0.236716719	0.485767499	0.487305	0.637692
KI	–0.416326607	0.511649697	–0.81369	0.436817
Other diseases	5.797262825	5.089930818	1.138967	0.284124
ABEP2015	1.337922656	4.406154348	0.303649	0.768298
Help from other caregivers	9.520891711	7.781361764	1.223551	0.252189
Total time as carer (YEARS)	–0.122479033	0.910787971	–0.13448	0.895986
Living in same dwelling	–11.26743373	10.76670289	–1.04651	0.322618
Weekly hours dedicated to patient care	–0.024971393	0.131735604	–0.18956	0.853862

P<0.05 (confidence interval of 95%); UPDRS: Unified Parkinson’s Disease Rating Scale/MMSE: Mini-Mental State Exam/KI: Karnofsky Index/ABEP2015: socioeconomic classification according to Brazilian Marketing Association version 2015.

The present study has some limitations. The sample was relatively small, although sufficient for statistical analysis by Pearson’s method and simple and multivariate linear regression with analysis of variance (ANOVA). However, a greater sample size would probably reinforce the statistical significance of some observed potential relationships, strengthening some observations. The Pearson’s correlation test precludes a conclusion on the causality between the variables. The method indicates only whether the dispersion of the increasing or decreasing values of each variable are correlated with each other and how strong the correlation is. The results of the study showed that the sole variable moderately positively correlated with ZBI score levels was UPDRS score, where higher UPDRS scores were associated with higher ZBI scores. Interestingly, the other severity scale used in the study (KI) also demonstrated a moderate but inverse correlation with ZBI scores, almost reaching statistical significance, given low KI scores represent worse condition of patients. However, when all the independent variables were analyzed by multivariate linear regression, none reached statistical significance in relation with the dependent variable (ZBI). However, based only on Pearson’s correlation method, it can be speculated that the severity of the disease can have a negative impact on the burden of caregivers.^24^ An attractive hypothesis is that the best PD treatment available, albeit pharmacological, non-pharmacological or even combined therapies, aimed at reducing UPDRS scores and keeping it low, might be the best strategy to promote a reduction in ZBI scores among caregivers. However, this is merely a speculation and multivariate linear regression analysis does not support this idea.

A weak non-statistically significant Pearson correlation, but also not supported by multivariate linear regression analysis, was found regarding cognitive status measured by the MMSE and ZBI scores. Other articles have observed that PD dementia and other non-motor symptoms were major negative factors in caregiver burden.^24^ No patient in the present survey was clearly demented according to scoring criteria of the MMSE, but it is important to reiterate that this method was considered to be a non-ideal tool for cognitive assessment in PD and was unable to detect many subtle subcortical, frontal and visuo-spatial deficits. However, in our “health care environment” it is widely used and extremely easy to apply. It is also noteworthy that other neuropsychiatric conditions, such as psychosis, hallucinations and delusions, commonly associated with PD, were undetectable by the MMSE. These factors might explain the weak negative correlation found.

Another possible correlation was explored, concerning whether the caregiver was alone in this role. A weak statistically insignificant correlation was present, suggesting that sharing care tasks may help decrease burden. Although obvious, we had expected a stronger and significant correlation. Again, the small number of participants may have contributed to this finding.

Because of the limitations outlined above, the authors are considering expanding the study with a larger sample to increase the power on multivariate regression statistical analysis and determine the predictive value of pharmacological, non-pharmacological and other combined therapy in reducing the burden of caregivers, since some interventions (or lack of them) can lead to greater functional decline for patients or decrease the burden of their caregivers.
